# Stem Cell-Based Trophoblast Models to Unravel the Genetic Causes of Human Miscarriages

**DOI:** 10.3390/cells11121923

**Published:** 2022-06-14

**Authors:** Tatiana V. Nikitina, Igor N. Lebedev

**Affiliations:** Research Institute of Medical Genetics, Tomsk National Research Medical Center, 634050 Tomsk, Russia; igor.lebedev@medgenetics.ru

**Keywords:** miscarriage, recurrent pregnancy loss, trophoblast, trophoblast stem cells, trophoblast organoid, extended blastocyst culture, aneuploidy, mosaicism

## Abstract

Miscarriage affects approximately 15% of clinically recognized pregnancies, and 1–3% of couples experience pregnancy loss recurrently. Approximately 50–60% of miscarriages result from chromosomal abnormalities, whereas up to 60% of euploid recurrent abortions harbor variants in candidate genes. The growing number of detected genetic variants requires an investigation into their role in adverse pregnancy outcomes. Since placental defects are the main cause of first-trimester miscarriages, the purpose of this review is to provide a survey of state-of-the-art human in vitro trophoblast models that can be used for the functional assessment of specific abnormalities/variants implicated in pregnancy loss. Since 2018, when primary human trophoblast stem cells were first derived, there has been rapid growth in models of trophoblast lineage. It has been found that a proper balance between self-renewal and differentiation in trophoblast progenitors is crucial for the maintenance of pregnancy. Different responses to aneuploidy have been shown in human embryonic and extra-embryonic lineages. Stem cell-based models provide a powerful tool to explore the effect of a specific aneuploidy/variant on the fetus through placental development, which is important, from a clinical point of view, for deciding on the suitability of embryos for transfer after preimplantation genetic testing for aneuploidy.

## 1. Introduction

The results of human reproduction are quite poor, with only around one third of conceptions progressing to a live birth [1,2,3]. Low reproductive efficiency is a characteristic feature of humans and is influenced by the low rate of conception (pregnancy occurs in only 18–28% of menstrual cycles in women engaging in regular sexual activity without the use of contraceptives [4]) and the high incidence of miscarriage (approximately 15% of clinically recognized pregnancies end in miscarriage, with the majority of these occurring in the first trimester of pregnancy). Human embryos have an extraordinary level of chromosomal abnormalities [5,6], which often do not significantly affect viability at the preimplantation stage. As a consequence, the main selection of embryos occurs either during implantation (manifested as low conception efficiency), or soon after implantation (manifested as miscarriage).

There is no doubt that the successful implantation and normal development of the placenta play crucial roles in the growth of the fetus in utero. Impaired placentation due to defective development or function of trophoblast cell lineages is considered one of the major underlying causes of pregnancy loss, especially in the first trimester. In human blastocysts, the morphological quality of the trophectoderm (TE), and not the inner cell mass (ICM), correlates with rates of ongoing pregnancy and miscarriage, which emphasizes the impact of normal trophectoderm lineage on pregnancy’s progression [7]. Although the causes of first-trimester loss are complex, miscarriage predominantly results from abnormal placentation and an impaired embryo–maternal interface [8,9].

A significant proportion of miscarriages (50–70%) are caused by chromosomal abnormalities; in addition, more data have appeared on the associations between pregnancy loss and copy number variations (CNVs), gene mutations, methylation abnormalities, and other aberrations [10,11,12,13,14,15]. One can suggest that the increasing number of studied families and gene variants/CNVs founded in early pregnancy loss highlights the need for their functional assessment in trophoblast development to determine their role in adverse pregnancy outcomes. It is necessary to understand how a specific abnormality (numerical, structural, monogenic, or epigenetic) leads to miscarriage—which member/pathway is disturbed under a specific pathology? The etiologic analysis of pregnancy loss is also necessary for medical management and the reproductive counseling of patients.

Data from preimplantation genetic testing (PGT) show that embryos with mosaic karyotypes can develop and produce chromosomally normal fetuses [16,17,18,19,20,21]. In some studies, it has been found that, in mosaic embryos, aneuploid cells are located predominantly in the trophectoderm/trophoblast lineage [22,23]. However, the aneuploid karyotype in the placenta per se can influence the development of the karyotypically normal fetus as a result of placental functional deviation [24]. An understanding is needed of how a specific aneuploidy can affect the phenotype of the fetus through placental development. For this, it is necessary to investigate the functional (and morphological) features of trophoblast cells with different variants of karyotypes and genotypes. Moreover, the study of the effects of specific aneuploidies on the development and function of the placenta is also important from a practical point of view for deciding on the suitability of mosaic embryos for transfer.

In recent years, due to the development of cell-based technologies, there has been a rapid increase in the use of various model systems for the study of the main functional epithelial component of the human placenta, the trophoblast. Different models provide unique tools for the identification of pathways involved in trophoblast lineage specification and differentiation. The derivation of human trophoblast stem cells (hTSCs) from both blastocyst-stage human embryos and first-trimester placentae, and the development of novel stem cell-based models of the trophoblast, provide a simplified system through which the complex processes underlying normal or defective placental development can be understood.

In this review, we will describe the different systems used for human trophoblast modeling, detailing the limitations and advantages of each approach for miscarriage research. We summarize current information about the findings obtained using trophoblast models for the study of placenta pathology, trophoblast development, and differentiation, associated with pregnancy loss. Furthermore, we will focus on the (stem) cell-based research of aneuploidy (partial or complete) in human trophoblast development. Differences between trophoblasts and ICM, in terms of the frequencies of aneuploid cells, mosaicism, and karyotype correction, will be discussed in the context of reproductive failure.

## 2. Genetic Causes of Miscarriage

Around 15% of clinically recognized pregnancies in humans result in miscarriage [25,26,27,28]. Although the majority of early pregnancy losses are sporadic, 1–3% of pregnant women suffer from recurrent pregnancy loss (RPL), defined as two or more consecutive pregnancy losses [28,29,30]. A strong pattern of miscarriage recurrence was found in a whole-population study, with the risk of recurrence being independent of maternal age, which implies that causes other than embryonic aneuploidy account for RPL [31]. Moreover, an increased probability of the same karyotype pattern (recurrent normal or recurrent abnormal) in multiple consecutive abortions was found for RPL patients [32,33,34]. In addition, women who miscarry may be more likely to have a family history of miscarriage [35]. The significance of genetic factors in miscarriage was proven by the six-fold increase in RPL prevalence among first-degree relatives of idiopathic RPL patients in comparison with the general population [36,37].

The most frequent cause of miscarriage is chromosomal abnormality. Almost 50 years of cytogenetic studies of spontaneous abortions have demonstrated abnormality rates of around 50–60% [10,38,39,40,41,42,43,44,45,46]. Trisomies are the most frequently detected anomalies (58–61%), followed by monosomy X (8–13%), polyploidies (2–13%), and structural anomalies (7–9%) [11,12,41]. Autosomal monosomies are rare in spontaneous abortion material (0.8–1.5%), and found predominantly in mosaic state [13,47,48,49].

DNA-based methods of molecular karyotyping, such as array comparative genomic hybridization (aCGH) and single-nucleotide polymorphism (SNP) microarray, have increased the diagnostic power, adding the detection of CNVs and uniparental disomy (UPD). The chromosomal abnormality rate detected by chromosomal microarray analysis (CMA) is similar to that obtained with conventional cytogenetics (approximately 50–60%), with 2–4.4% CNV and 0.25–0.5% UPD [13,14,50].

Karyotype evaluation of first-trimester miscarriages using next-generation sequencing (NGS) found a total abnormality rate of 63%, with 73% numerical variants (including 60% aneuploidies, 7% polyploidies, and 5.5% mosaicisms); the other 27% of cases showed microstructural variants [15].

Overall, cytogenetic studies find causes in 50–60% of embryo losses, while the reason for the miscarriage in most other cases often remains unclear.

In euploid abortions, using molecular techniques such as aCGH, SNP array, and NGS, the possible candidate genes associated with RPL have been revealed, including genes involved in the immune response, coagulation, metabolism, angiogenesis, cell division, cilial function, and fetal movement [51,52,53]. The identification of genes that are causative of or predisposing to pregnancy loss is of significant impact for patient counselling and treatment, though most of these genes show moderate associations with RPL and have been described in only small case–control studies, as reviewed by [54,55,56].

In total, mutations in candidate genes responsible for recurrent embryonic loss were found in up to 60% of cases [56]. Such studies provide the possibility of understanding the biological pathways that can cause pregnancy loss. Now, the increasing number of studied RPL families produces an escalating number of CNVs/gene mutations/variants found in pregnancy loss, which need to be investigated to determine their role in adverse pregnancy outcomes. Since current methods of genome and transcriptome analysis provide an enormous bulk of complex data, the molecular aspects of miscarriages must be fully understood for accurate interpretation of the data. Contemporary cell-based techniques for in vitro trophoblast derivation offer the possibility of the functional assessment of specific chromosomal abnormalities, CNVs, or gene variants in human pregnancy loss.

## 3. Placental Defects Are the Main Cause of First-Trimester Human Miscarriage

The trophoblast lineage consists of the TE, cytotrophoblast (CTB), syncytiotrophoblast (STB), and extravillous trophoblast (EVT). The TE, the outer layer of the blastocyst, is the precursor of all trophoblast cell types and contributes significantly to implantation. Implantation of the blastocyst at day 5−6 is the beginning of human placental development, when the TE fuses to the primary syncytium and the conceptus intrudes into the decidua. The transition from the TE to the CTB is thought to represent implantation into the uterine wall. Following implantation, the placenta forms a system of branching villi attached to the decidua and, by week 3 post-conception, all the trophoblast subtypes are present: the villous cytotrophoblast (VCT) that differentiates into two lineages, STBs in floating villi and EVT in anchoring villi. Early in gestation, the placental villi are covered by VCTs with overlying STB, which is a mitotically inactive multinucleated layer and the major area of maternal–fetal nutrient transport, gas exchange, and pregnancy hormone secretion. VCTs are mononuclear cells that form an epithelial layer separated by a basement membrane from the underlying stromal core. Cell islands of EVTs bud out of villi and, when they contact the decidua, invade from the villi into the endometrium, anchoring the placenta to the uterus and later transforming the maternal spiral arteries. Thus, during the initial stages of implantation, the trophoblast cells produce substances and mediators that allow for adhesion and invasion into the uterine wall, alter the maternal immune cell phenotype to prevent embryo rejection, and generate hormones to maintain pregnancy progress [57,58,59]. Defects in these processes are the most common causes of early pregnancy loss [9].

In a recent study in mice, Hemberger’s group demonstrated that the vast majority (68%) of gene knockouts that cause fetal developmental defects and embryonic lethality are linked to placental dysfunctions, suggesting that an abnormal placenta is often an unappreciated cause of embryonic lethality [60]. Early lethality (mouse embryonic days 9.5–14.5) was found to almost always be associated with severe placental malformations [60]. Interestingly, an analysis of mutant trophoblast stem cells and conditional knockouts showed that a significant number of factors that cause mouse embryonic lethality have a primary gene function in trophoblast cells [60]. Placental insufficiency in rats that have deep hemochorial placentation also negatively affects fetal survival and growth [61]. In humans, the results of recent large-scale genetic association analyses with 69,054 cases from five different ancestries for sporadic miscarriage show that miscarriage etiopathogenesis is partly driven by genetic variation, potentially related to placental biology [62].

Besides miscarriage, the abnormal development or function of the placenta underlies many other complications of pregnancy, including preeclampsia (PE), fetal growth restriction (FGR), preterm birth (PTB), and fetal malformation [63,64,65,66]. Although pregnancy complications such as FGR, PE, and PTB occur during the second and third trimesters, the primary mechanisms of these diseases have been implicated in the early stages of intrauterine development [67,68,69]. In a comprehensive population study, it was shown that certain other pregnancy outcomes (preterm delivery, stillbirth, caesarean section, etc.) cluster with the risk of miscarriage, suggesting that these outcomes might share underlying causes [31].

Furthermore, defective placentation could lead to deviation in fetal development, leading to poor postnatal health or susceptibility to diseases in adulthood [70,71]. Importantly, in mice, placental defects correlate strongly with abnormal brain, heart, and vascular development [60]. In humans, some studies indicate a link between placental biology, early-life complications, and schizophrenia [72], or between a history of maternal spontaneous abortion and offspring developmental disorders, including intellectual disability [73,74,75].

## 4. Stem Cell-Based Trophoblast Models

Because studies of early human placental development were restricted by limited access to primary tissues and ethical issues, animal models (mice in particular) were used to investigate trophoblast lineage specification and function. Although both mouse and human placentae are discoid in shape with a hemochorial gas–nutrient exchange, mouse model systems do not completely mimic the human placenta, with essential differences including gestational length, litter size, embryo architecture, trophoblast cell types, and tissue organization [76]. Furthermore, human and mouse TE establishment differ in terms of the expression of some crucial genes during the early development of the two species, as surveyed by [77,78,79,80,81,82]. Efforts to obtain human trophoblast stem cells (hTSCs) from blastocysts using culture conditions analogous to murine TSCs have not been successful [83]. Moreover, mice lack some genes critical for the development of human trophoblasts, such as gene *NLRP7*, which does not allow for the simulation of appropriate disorders in mice [84].

Other human trophoblast studies relied on cancer cell lines derived from choriocarcinoma and immortalized cell lines. However, there were multiple drawbacks. Carcinoma cells (such as BeWo, JEG-3, and JAr cells) do not perfectly recapitulate the multipotent human trophoblast, and show an abnormal gene expression profile. Several cell lines of placental origin, such as HTR8/SVneo, TEV-1, ACH-3P, SGHPL-5, and HIPEC65, have been immortalized from isolated first-trimester EVTs by genetic manipulation [85,86,87,88], but these lines are unreliable for the investigation of normal placenta physiology, as they have varying differentiation ability, transcriptomes, and other properties compared with primary human trophoblasts [79].

The substantial differences between human embryos and mouse models or choriocarcinoma/immortalized cell lines emphasize the necessity of working with human stem cells and organoid models to understand human placental development.

In recent years, studies of trophoblast lineage specification and function have reached a new milestone due to a combination of cell-based technologies, reprogramming, and whole-transcriptome analysis, especially single-cell RNA-seq. This combination of advances has led to a rapid increase in the possibility of modeling the trophoblast and its specific cell types. Recently, there has been considerable growth in studies that elaborate on the various modeling methods and approaches to obtaining the trophoblast and its derivatives. This creates unprecedented opportunities to study the causes of reproductive problems in humans, including miscarriage.

The use of cell-based technologies for modeling the trophoblast or placental dysfunction associated with miscarriage is possible in two main ways: direct and mediated. The direct way is to use placental tissue from miscarriages with a genetic abnormality to extract trophoblast stem cells, followed by differentiation to specialized cell types and the analysis of the cell phenotype, gene expression, or protein function to determine the relationship of a specific chromosomal or single gene disorder with placental dysfunction and embryo lethality. This may be hampered by not knowing the cause of the pregnancy loss at the moment of abortion.

Other strategies use the intermediate stage of pluripotent stem cell (PSC) derivation. This may involve patient-specific PSCs with a known abnormality or the introduction of a mutation into normal hPSCs using genome editing methods, with subsequent derivation of proliferative-capable trophoblast stem cells (TSC), followed by differentiation into specific cell types of the placenta. In this case, the impossibility of obtaining “true” trophoblast stem cells from primed iPSCs could be a problem; however, recent studies demonstrate the possibility of iPSC-based TSC derivation, implementing such a method for pregnancy loss research [89,90].

**Figure 1:**
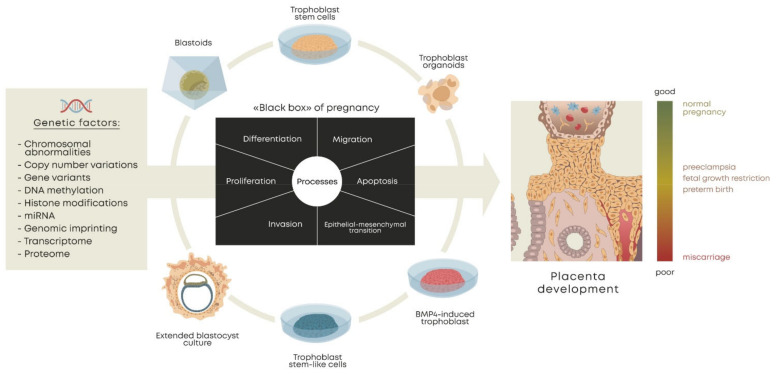
Stem cell-based technologies for the study of human trophoblasts and placental development. Genetic factors affect proper human embryo development and pregnancy success. Many processes in early development are hidden in the “black box” of human implantation [1]. Stem cell-based technologies can be applied to study gene functions or manifestations of chromosomal abnormalities in human trophoblast lineage. This opens up the possibility of an experimental study of the processes of early placental development and the underlying molecular mechanisms of the pathogenesis of pregnancy complications, among which miscarriage is the most common.

The main approaches to the derivation of trophoblast cells for modeling are BMP4-induced trophoblast-like cells (terminal trophoblast-like cells); self-renewing trophoblast stem cells (TSC, true hTSCs), isolated from the trophectoderm of human blastocysts and first-trimester placentae; trophoblast stem-like cells (TSLC), derived from naïve/expanded/primed PSCs; trophoblast stem cells induced by the ectopic expression of transcription factors (iTSCs); 3D culture models; blastoids (artificial embryos generated using TS and ES cells together); and extended blastocyst cultures (Figure 1).

It is necessary that the resulting cells recapitulate the hallmarks of the corresponding cell phenotypes: the expression of specific transcription factors, the long-term self-renewal ability, and the potential to differentiate into syncytiotrophoblasts and extravillous trophoblast cells. Criteria that are characteristic of primary first-trimester trophoblasts have been proposed: the expression of genes highly expressed in trophoblasts, such as TFAP2C, GATA3, and KRT7 (cytokeratin); a HLA class I expression pattern (either HLA null (in VCT and STB) or HLA-G+, HLA-C+ but HLA-A− and HLA-B− (in EVT)); very high expression of the C19MC (chromosome 19 microRNA cluster); and hypomethylation of the ELF5 promoter [91]. This panel can be used to define cells as characteristic of early trophoblasts. Different approaches to modeling trophoblast cells produce cell types that meet these criteria with varying degrees of conformity.

## 5. Methods for Derivation of Cells and Organoids for Trophoblast Modeling

### 5.1. BMP4-Induced Trophoblast-Like Cells (Terminal Trophoblast-Like Cells)

Human pluripotent stem cells (hPSCs) were first reported to have trophoblast differentiation potential in 2002, when human chorionic gonadotropin (hCG)-secreting multinucleated trophoblasts were identified in cultures supplemented with bone morphogenetic protein-4 (BMP4) [92]. Nowadays, the conversion of hPSCs to trophoblast-like cells by treatment with BMP4, alone or in combination with small molecules, named BAP (BMP4, A83–01 (Activin/NODAL/TGFB pathway inhibitor) and PD173074 (FGFR1 inhibitor)), is a common method [93,94,95,96]. Resulting cells express trophoblast-specific markers, including HLA-G, show invasive capacity, and have the ability to form EVT-like cells and secrete placental hormones, such as human chorionic gonadotropin (hCG), progesterone, and placental lactogen [95]. Horii and colleagues showed that CTB-like cells have the potential to differentiate into a mixture of terminally differentiated hCG-secreting multinucleated STB-like cells and invasive HLA-G+ EVT-like cells (bipotency) [96].

Whether the cells obtained from BMP4-treated hPSCs actually correspond to the trophoblast is under discussion. It was supposed that these cells have not fully differentiated to trophoblasts on the basis of four criteria: a largely hypermethylated ELF5 promoter, lack of expression of the chromosome 19 miRNA (C19MC), a particular profile of HLA-class I molecules, and a discrepancy in global gene expression profiles from primary tissues [91]. Because the trophoblast and amnion share many common genes—for example, the expression of hCG and HLA-G—some researchers suggest that BMP4-induced trophoblast-like cells have gene expression patterns more similar to the amnion [97,98] or correspond to extra-embryonic mesoderm derivatives [99,100]. A recent study from the Parast laboratory confirms that BMP4 treatment alone induces a mixture of trophoblast and mesoderm fates in primed hPSCs [101].

Another opinion is that the hPSC-derived trophoblast represents a short-lived form of trophoblast that emerges as the embryo begins to implant during the second week of pregnancy [102,103]. Comparison of STBs generated from BAP-treated hESCs and from blastocysts, trophoblast stem cells, and placentae found that both syncytial cells are trophoblasts, with the potential to transport a wide range of solutes and synthesize placental hormones [104,105], but hESC-generated STBs represent the primitive syncytium encountered in early pregnancy, soon after the trophoblast invades the uterine wall [104].

To prevent mixed phenotype cultures, inhibition of the ACTIVIN-A/NODAL/TGFB and FGF2 signaling pathways was used, resulting in cultures that were 80–100% trophectoderm or trophoblast-like, with undetectable levels of the mesodermal marker Brachyury [96]. Another approach is the purifying of cells derived from BMP4-treated iPSCs using flow cytometry with pan-TB marker keratin 7, and the transcriptome of the resulting KRT7+ cell population is similar to human placental tissue [106].

Another limitation of the BMP4-induced model is the lack of self-renewal, with short-time proliferation and quick differentiation. Nevertheless, BMP4-induced trophoblast-like cells have many advantages over other models, since iPSCs can be used as a cell source, offering the possibility to generate patient-specific trophoblast-like cells or to perform genetic manipulations [107]. Thus, models of miscarriage-related trophoblast differentiation defects were derived for trisomy 21, monosomy X, and translocation t(11;22) [96,103,108].

As shown earlier, placentae with trisomy 21 have prolonged maintenance of a continuous CTB layer into the second trimester and abnormalities of STB formation [109]. Horii and co-workers used BMP4-derived trophoblast cells from two trisomy 21 hPSC lines, one ESC line and one iPSC line; as a control in each case, a subclone of the same cell line without an extra copy of chromosome 21 was applied. Trisomy 21 hPSC showed a delay in the induction of the trophoblast lineage, as measured by surface EGFR expression, a slower decrease in the expression of the pluripotency factor POU5F1/OCT4, and an exaggerated induction of CDX2. CTBs derived from T21-hPSCs showed a lower fusion index, increased hCG secretion, and altered expression of the transcripts of the hCG components CGA and CGB. Thus, the differentiation of trisomy 21 hPSCs recapitulates the delayed CTB maturation and blunted STB differentiation seen in trisomy 21 placentae [96].

Monosomy X is one of the most frequent miscarriage causes, estimated to account for 6–11% of all pregnancy losses [110,111]. The 45, X karyotype was modeled by BAP-treated iPSC with monosomy X and isogenic euploid male and female controls in order to test how X/Y-linked gene dosage impacts trophoblast development. While isogenic panels trigger a GATA2/3- and TFAP2A/C-driven trophoblast gene circuit irrespective of karyotype, differential expression implicates monosomy X in altered levels of placental genes, and in the secretion of placental growth factor (PlGF) and human chorionic gonadotropin (hCG). These results suggest that monosomy X may skew the trophoblast cell type composition, and that the pseudoautosomal region likely plays a key role in these changes. This system provides the first direct assessment of how monosomy X may impact human trophoblast-relevant gene networks [103].

Repeated miscarriages are often in carriers of the balanced translocation due to the high risk of creating unbalanced gametes. To study the biological basis of miscarriage in cases t(11;22), the most common reciprocal translocation in humans, ESCs with t(11;22) were differentiated into trophoblast-like cells using BMP4 treatment. Trophoblast-like cells with translocation displayed reduced and delayed secretion of β-hCG concomitant with impaired expression of the trophoblastic genes CDX2, TP63, KRT7, ERVW1, CGA, GCM1, KLF4, and PPARG compared to the control [108]. Subsequently, trophoblast vesicles were created, and the number of vesicles attached to endometrial cells was significantly lower. Correspondingly, the invasiveness of trophoblast-differentiated cells with translocation was also significantly lower compared to the control [112]. These results may explain the implantation failure in couple carriers of t(11;22), and demonstrate that trophoblast-differentiated hPSCs could be a valuable in vitro human model for studying the mechanisms underlying miscarriage.

Trophoblast-like cells, derived from BMP4-treated triploid ESC (the product of a tripronuclear zygote), showed morphology, gene expression, and secretion of hCG, similar to a diploid hESC line [113]. Previously, it has been reported that triploid hESCs have an ability to spontaneously differentiate to the trophoblast lineage, with hCGs detected in the conditioned media at a level of more than 35 mIU/mL [114]. However, these ESC-derived trophoblast-like cells are insufficiently characterized to serve as a model for triploid trophoblasts and need further elaboration.

Recently, two groups implemented trophoblast differentiation of iPSCs for the simulation of another complex pregnancy disorder, preeclampsia, and identified abnormalities in oxygen response mechanisms concomitantly with blunted STB formation and maturation [115,116]. The finding that two different cohorts of preeclampsia iPSC-derived trophoblasts show abnormal responses to changes in oxygen tension suggests that this model can be used to recapitulate this complex pregnancy disorder. Burton and Jauniaux, in 2004, suggested that “miscarriage, missed miscarriage, and early and late onset preeclampsia represent a spectrum of disorders secondary to deficient trophoblast invasion” [117]. Thus, reduced trophoblast invasion under high O_2_ conditions, demonstrated in cell-based models of preeclampsia by two groups [115,116], is in accordance with this hypothesis, as well as the results of Shpiz and colleagues for translocation t(11;22) [112].

Very interesting results were recently obtained by Alici-Garipcan and co-workers in a study of iPSC-derived trophoblasts from a patient with a recurrent complete hydatidiform mole—a gestational trophoblastic disease resulting in the hyperproliferation of trophoblast cells and the absence of the embryo itself [84]. Patient-specific iPSCs carrying the compound heterozygous variant of gene NLRP7 were BAP-differentiated, and whole-transcriptome profiling showed that impaired NLRP7 expression results in the precocious downregulation of pluripotency factors and the activation of trophoblast lineage markers, and promotes the maturation of differentiated syncytiotrophoblasts: STB-related genes such as ERVW-1 (Syncytin) were remarkably enriched compared to the control. Surprisingly, trophoblast differentiation in NLRP7-mutant iPSCs does not require exogeneous BMP4, which suggests that the patient’s cells may undergo excessive trophoblast differentiation due to dysregulation of BMP4 signaling [84]. These results are in accordance with the recently described finding that BMP4 and the GATA3 axis are regulators of commitment to exit from pluripotency and cell fate decision in early human embryo development [118].

Recently, a study was published wherein BMP4-stimulated hESCs were used to test the lineage-specific behavior of aneuploid cells in early human embryogenesis [119]. Previously, it was shown that hESCs cultured in micropatterns reach a cell density equivalent to that observed in the pre-gastrulating human epiblast [120]. Addition of BMP4 triggers pluripotency exit and lineage commitment with radially organized germ layers, where the TE and amnion are on the outside, followed by the endoderm, mesoderm, and ectoderm towards the center of the colony [121,122]. Using such self-organized structures, named “gastruloids,” Yang and colleagues showed that, in the extra-embryonic territory, cells express markers of the early trophectoderm (for example, CLDN4, SLC7A2, and TACSTD2) similarly to day 5 embryos. The authors stimulated aneuploidy occurrence by reversine (an inhibitor of monopolar spindle 1 kinase that inactivates the spindle assembly checkpoint), causing defects in chromosome segregation. As a result of BMP4 treatment, aneuploid gastruloid colonies predominantly produced GATA3+ cells due to the prevalent survival of extra-embryonic tissue [119].

### 5.2. “True” Human Trophoblast Stem Cells (hTSCs)

The first derivation of self-renewing TSCs harboring bipotency was implemented from human blastocyst trophectoderm and first-trimester placental isolates in 2018 [123]. Both derivations were achieved by the manipulation of multiple signaling pathways (activation of the epidermal growth factor (EGF) and Wingless/Integrated (Wnt) signaling pathways, along with inhibition of the transforming growth factor beta (TGFB) pathway) combined with histone deacetylase (HDAC) inhibitors and Rho-associated protein kinase (ROCK) inhibitor treatment. This technique makes it possible to obtain self-renewing cytotrophoblast cells that can give rise to both STBs and EVTs (bipotency). Human TSCs were able to be maintained in an undifferentiated state for more than 80 passages and met the four criteria for trophoblast cells [91]. Human TSCs injected into mice mimicked trophoblast invasion during implantation, and their molecular, transcriptomic, and epigenetic characteristics were similar to both primary trophoblast isolates and the day 10/12 ex vivo cultured human trophoblast [123,124].

The culture system, named hTSC media or Okae media, was optimized for maintaining specific conditions and comprised CHIR99021 (a Wnt activator), EGF, Y27632 (ROCK inhibitor), A83-01, SB431542 (TGFB inhibitor), and valproic acid (VPA) (a histone deacetylase inhibitor) or trichostatin A (TSA) or suberoylanilide hydroxamic acid (SAHA). This medium is widely used for cultures of trophoblast stem cells, including trophoblast stem-like cells derived from hPSCs with different techniques.

Although hTSCs provide a good in vitro model system of the placenta and reliable protocols to differentiate them into STBs and EVTs are available, their use is limited by the difficult access to blastocysts or primary CTBs from the first-trimester placenta due to ethical concerns. Nevertheless, this method could become quite feasible for the study of miscarriage because first-trimester pregnancy losses are the most common, offering the possibility of obtaining placental tissues followed by self-renewing trophoblast stem cell derivation.

This approach was used in recent studies of recurrent pregnancy loss [8,125], where TSCs were derived from first-trimester placental tissues. Saha and colleagues found an association of idiopathic recurrent pregnancy loss with the expression of transcription factor TEAD4, an effector of the Hippo signaling pathway [8]. Tead4 is selectively expressed in trophoblast progenitor cells of early postimplantation mouse embryos, and the loss of Tead4 in the postimplantation mouse leads to embryonic lethality before embryonic day 9.0, equivalent to the first trimester of human gestation [126]. This gene was found to control the self-renewal and stemness of trophoblast progenitors in postimplantation human embryos to ensure placentation and the progression of pregnancy [8].

Patient-specific TSCs were established from CTBs of 22 patients with RPL, using modified Okae media. Seven RPL placentae showed a prominent defect in placental villi formation with a defective CTB/STB bilayer and a strong reduction in TEAD4 expression in VCTs and column CTBs [8]. TSC lines were established from only five of these seven defective placentae, and, despite being euploid, these TSC lines had extremely slow proliferation and reduced expression of TEAD4 mRNA as well as protein expression. They also showed a higher propensity to lose the stem state cellular morphology and were compromised in TSC organoid formation. Thus, impairment of the Hippo signaling pathway disturbs the balance of self-renewal vs. differentiation in idiopathic RPL placentae [8].

It was found that neddylation inhibition in recurrent spontaneous abortion trophoblasts causes the cytoplasmic retention of free NEDD8 and p21 accumulation [125]. TSCs were used to model trophoblast differentiation and it was demonstrated that neddylation inhibition significantly hindered EVT differentiation by downregulating EVT markers HLA-G, MMP2, and ITGA1 and upregulating the CTB marker CDH1 during the differentiation process, thereby causing proliferation restriction and plasticity impairment [125].

These findings show that hTSCs open up wide opportunities for the study of pathological variants and modeling of impairments that could be a molecular cause for early human pregnancy loss [8,125]. At the same time, an attempt to use the differentiation of primary hTSCs to EVTs in order to evaluate the development of polyploidy in in-vitro-differentiated EVTs showed that this method does not recapitulate the polyploid phenotype seen in primary EVTs. It is possible that, in vivo, EVTs receive additional signals from their environment that lead to polyploidization [127].

### 5.3. Trophoblast Stem-Like Cells (TSLC)

Several methods for the derivation of cells, analogous to hTSCs, have been reported in recent years from different starting cell types, including naïve hPSCs (which correspond to the preimplantation blastocyst), primed hPSCs (which correspond to the postimplantation epiblast), and extended or expanded hPSCs (intermediate state).

Recently, multiple research groups have reported the successful conversion of naïve hPSCs into cells that exhibit cellular and molecular phenotypes of human trophoblast stem cells, including unlimited replication [97,98,124,128]. Collectively, this type of cell can be called trophoblast stem-like cells (TSLC). Naïve hPSCs can directly give rise to human TSLCs when cultured in Okae media, as confirmed by morphological, molecular, and transcriptomic criteria [124]. Naïve hPSC-derived TSLCs can be maintained for over 20 passages without bipotency loss, and they show overall gene expression signatures similar to hTSCs as well. Modest differences were observed between transdifferentiated and placental hTSCs, most notably in the expression of certain imprinted loci. It has been shown that naïve iPSCs can permit the genome-wide loss of DNA methylation [129], which may violate the expression of imprinted genes, which are important for extra-embryonic development [130,131]. Abnormal imprinting in the human placenta is associated with various disorders, including miscarriage [132,133]. Taking into account that differentially methylated regions are maintained in a placenta-specific manner, the aberrant DNA methylation in naïve iPSC-derived TSLCs can restrict their application in the modeling of pregnancy complications.

The latest study showed that human naïve epiblast stem cells readily produce an extra-embryonic trophectoderm lineage, and ERK and TGFB inhibition of naïve hESCs results in differentiation to trophoblast-like cells [97]. Interestingly, ICMs from expanded human blastocysts efficiently regenerated the trophectoderm. Thus, in humans, retained trophectoderm potential is an integral feature of pluripotency that confers higher plasticity. Although human trophoblasts arise as TE at the morula stage, single-cell RNA sequencing data suggest that the determination of the fate of cells might be late [134].

Recently, some other means of differentiating hPSCs into trophoblast stem-like cells have been developed. One is based on a new type of stem cell, expanded-potential stem cells (EPSCs). Using a novel combination of transcription factors, human ESCs and iPSCs can be converted, or somatic cells directly reprogrammed, into human EPSCs, which in turn can be efficiently differentiated into trophoblasts [135].

In contrast to the previous failures to generate self-renewing hTSLCs from primed hPSCs using BMP4 [93,94,96], several independent groups have demonstrated the successful conversion of primed hESCs to hTSLCs. One group showed that the culture of iPSCs on a nickel micromesh with triangular shapes for 30 to 50 days results in cysts, and the cells derived from the reseeded cysts have the characteristics of hTSCs [136]. These cells, induced using a micromesh technique without any chemical stimulation, proliferated for over 205 days, showed high expression levels of TSC-specific genes and bipotency.

More recently, another study showed that primed hPSCs can be differentiated to hTSLCs in chemically defined media containing the phospholipid sphingosine 1-phosphate (S1P) together with BMP4 and SB43154 [89]. Initial S1P treatment of hESCs leads to the derivation of CTB-like cells with the potential for the subsequent formation of both EVT- and STB-like cells, so S1P, Rho/ROCK signaling, and YAP are necessary for trophoblast differentiation from hESCs.

Tietze and colleagues applied another approach to identify conditions that efficiently drive the specification of primed iPSCs to TSLCs. Temporal single-cell RNAseq was used for the definition of the molecular changes associated with TSC derivation under three separate conditions: (i) BMP4, (ii) BMP4 and inhibition of WNT, and (iii) activation of EGF and WNT, and inhibition of TGFB, HDAC, and ROCK signaling (conforming with Okae media). It was found that BMP4 gives rise to mesenchymal cells, while TSC conditions without exogenous BMP4 generate a stable proliferating cell type that is highly similar to six-week placental cytotrophoblasts. TFAP2A and ESRRG are central genes that program the specification of primed iPSCs to TSLCs without transitioning through a naïve state [100]. iPS-derived TSLCs were capable of self-renewal for at least 30 passages, differentiating into syncytial cytotrophoblasts and villous cytotrophoblasts and generating villi-like structures in low-oxygen conditions [100].

More recently, Wei and co-workers described the successful derivation of TSLCs from primed hPSCs with culturing in a hTSC medium containing l-ascorbic acid, epidermal growth factor (EGF), glycogen synthase kinase 3 (GSK3) inhibitor CHIR99021, TGF-β inhibitors A83-01/SB431542, histone deacetylase inhibitor valproic acid (VPA), and rho-associated, coiled-coil-containing protein kinase (ROCK) inhibitor Y27632 [90]. Interestingly, BMP4 treatment was found to substantially enhance the efficiency of generating hTSLCs from primed PSCs. Resulting cells were similar to blastocyst-derived hTSCs in terms of the morphology, proliferation, and differentiation potential, with high expression of TSC markers and lacking expression of pluripotent markers. In addition, the promoter of ELF5 was hypomethylated and HLA class I molecules were not expressed in these cells at passage 10. Furthermore, the hTSLCs exhibited low apoptosis, high proliferation ability, and colony formation from single cells. These data indicate that hTSLCs generated under TSC medium containing BMP4 also have trophoblastic features and stem cell properties [90]. Consistent with the low density of H3K27me3 in primed hPSC-derived hTSCs, it was shown that knockout of H3K27 methyltransferases (EZH1/2) increases the efficiency of hTSLC derivation from primed hPSCs [90].

Efficient derivation of hTSCs from primed hPSCs provides a simple and powerful model to understand human trophoblast development. Undoubtedly, additional functional tests are still required to confirm the true nature of many of the hTSLC lines produced by different methods. However, the results already available indicate that different culture conditions can be used to convert hPSCs, naïve or primed, to hTSLCs, suggesting that there may be multiple molecular paths for the conversion of hPSCs toward hTSLCs.

### 5.4. Induced Trophoblast Stem Cells (iTSCs), TF-Mediated Conversion Models

Another means to establish TSCs is the reprogramming of somatic cells into induced trophoblast stem cells (iTSCs) by the ectopic expression of transcription factors [137,138]. Single-cell transcriptomic analysis revealed that, during reprogramming into primed and naive pluripotency, a subpopulation of cells that enter a trophectoderm-like state emerged. Furthermore, this trophectoderm-like state could be captured with hTSC culture conditions, which permit the derivation of iTSCs [138]. The induced trophoblast stem cells are molecularly and functionally similar to the trophoblast stem cells derived from human blastocysts or first-trimester placentae [123]. Remarkably, whereas mouse iTSCs can be derived by the overexpression of trophoblast-specific TFs EOMES, GATA3, TFAP2C, and MYC in mouse fibroblasts [139,140], human iTSCs were established by reprogramming somatic cells with induction with Yamanaka’s cocktail (OCT4, KLF4, SOX2, and MYC), coupled with hTSC culture conditions [137,138]. However, non-integrating viral expression of transcription factors TFAP2C, TEAD4, CDX2, ELF5, and ETS2, as shown, efficiently generated TSC-like cells from the term placenta villous cytotrophoblasts (vCTBs) [141]. The iTSCs express TSC markers such as GATA3, TEAD4 and ELF5, are capable of differentiation into both EVTs and STBs, and can be passaged indefinitely without a slowing of growth. The transcriptome profile of these cells closely resembles the profile of hTSCs isolated from first-trimester placentae [141].

The coexistence of primed-like, naïve-like, and TE-like cells revealed during reprogramming in the fibroblast medium without exposure to any pluripotent or trophoblast media suggests that the OKSM combination can induce human fibroblasts to acquire pluripotent and trophoblast states without ectopic expression of trophoblast-specific transcription factors. This surprising result suggests that at least a small number of cells harbor active trophoblast-specific gene expression during the intermediate stage of reprogramming. TSC culture conditions may stabilize hTSC-specific gene expression programs by repressing the upregulation of other lineage-specific genes [138]. The developmental stage of hiTSCs resembles postimplantation NR2F2+ cytotrophoblasts on days 8–10 [137]. The fact that the OSKM system, largely accessible to researchers, is not restricted to embryonic lineages, but also supports the trophoblast fate, opens up an opportunity for the parallel generation of isogenic hiTSCs and hiPSCs, which could greatly benefit the study of placenta-related diseases, including miscarriage.

### 5.5. 3D Culture Models

Cells cultured on two-dimensional (2D) surfaces do not always accurately recapitulate authentic tissue environments, where cells are spatially surrounded by other cells in three dimensions. Advances in culture conditions, from two-dimensional monolayer culture to three-dimensional (3D) systems, have led to the development of trophoblast organoids, enabling us to study human trophoblast function in the context of a more physiologically accurate environment. In 2018, two articles were published based on the results of the study of trophoblast organoids (TB-ORGs). In the first paper, Haider and colleagues used EGF, the TGFB signaling inhibitor A83-01, the BMP signaling inhibitor Noggin, and the activators of Wnt signaling, R-spondin, CHIR99021, and prostaglandin E2, and described the generation of trophoblast organoids from purified first-trimester CTBs. Molecular analyses revealed that the CTB organoid cultures (CTB-ORGs) expressed markers of trophoblast stemness and proliferation and were highly similar to primary CTBs in terms of the global gene expression [142]. Under self-renewing conditions, the organoids were composed of CTBs (outside) and STBs (inside) that were spontaneously differentiated from CTBs, whereas the withdrawal of factors for self-renewal (Wnt stimulators) induced trophoblast outgrowth, expressing the EVT progenitor marker NOTCH1, and provoked the formation of adjacent, distally located HLA-G+ EVTs. Organoids were passaged approximately every 14 days and could be expanded for more than five months, but after passage 13, growth rates decreased, which indicates their incomplete (compromised) self-renewing ability.

Another group also established genetically stable trophoblast organoids that can differentiate into both STBs and EVTs from EPCAM-positive proliferative trophoblasts, using EGF, FGF2, A83-01, CHIR99021, and R-spondin [143]. TB-ORGs, growing in growth factor-reduced Matrigel, mimic the in vivo structure of the human placenta, forming villous-like structures with an inverse organization, where the basement membrane is located outside the organoids and multinuclear, E-cadherin-negative cells reside in the central cavity. These organoids secrete placenta-specific hormones and growth factors, and can further differentiate into EVTs [143]. A recent study of this group delineates the optimal composition of the trophoblast organoid medium (TOM) that contains EGF, hepatocyte growth factor (HGF) and FGF2 (MAPK activators), CHIR99021 and R-spondin-1 (WNT activators), Y-27632 (a ROCK inhibitor), prostaglandin E2 (PGE2) (a cAMP/AKT activator), and A83-01 (a TGFB inhibitor). Under the above conditions, organoids form villous-like structures containing SCTs and VCTs and can be propagated for up to one year [144]. The authors found that organoids resembled the villous placenta in terms of their transcriptomes and the production of placental hormones. In summary, these studies suggest that the activation of Wnt and EGF signaling and inhibition of the TGFB pathway could be sufficient for the derivation and long-term expansion of human TB-ORGs. TB-ORGs consist exclusively of trophoblast cells, allowing researchers to study the discrete steps of placental development in a simplified system.

Most recently, the derivation of trophoblast organoids from TSCs [8] and naïve hPCs [145] was also reported. Similar to CTB-derived organoids, hTSC organoids had villous-like structures and showed an inverse organization of undifferentiated and differentiated cells. The scRNA-seq showed that human TSC organoids reproduce progenitor CTB renewal and differentiation, and identified the basal cell adhesion molecule (BCAM) as a primitive progenitor marker. BCAM enrichment or gene silencing resulted in enhanced or diminished trophoblast organoid growth, respectively [146].

Interestingly, control human TSCs formed large organoids with prolonged culture and could be dissociated and reorganized to form secondary organoids, indicating self-renewing ability, whereas some TSC lines, derived from RPL placentae with reduced expression of TEAD4, were inefficient at forming a TSC organoid structure or formed much smaller organoids, suggesting a significant role of the CTB progenitor gene *TEAD4* in human placenta development [146] and idiopathic RPL origin [8]. Similarly, using TB-ORGs, the pivotal role of YAP–TEAD4 complexes in trophoblast proliferation and expansion was demonstrated, activating cell cycle and stemness genes and suppressing trophoblast cell fusion [147].

### 5.6. Artificial Embryos Generated Using TS and ES Cells Together (Blastoids)

Until recently, modeling the human embryo with stem cell-derived structures was based on ESCs cultured without extra-embryonic stem cells, so models of human pre-gastrulation including extra-embryonic tissues were not reported before 2021. Successful derivation of mouse blastocyst models, called blastoids, used two different approaches: assembling pre-established stem cell lines [148,149,150], or differentiating extended or expanded pluripotent stem cells into blastocyst-like structures [151]. Progress in the derivation of human trophoblast stem cells from various types of pluripotent cells opened up the possibility of generating artificial embryo-like structures for humans as well. In 2021, several independent teams published methods to obtain human blastocyst-like structures in vitro [152,153,154,155,156,157].

Based on the previously demonstrated fact that naïve human ES cells are competent for differentiation into both embryonic (epiblast) and extra-embryonic (trophectoderm and hypoblast) lineages [97,124], naïve hPSC lines were differentiated into cells mimicking the main cell types composing blastocysts. Using a three-dimensional culture system, as well as sequential stimulation of the cells with growth factors, inhibitors, and cytokines promoting the specification of epiblast (EPI) and TE lineages, blastocyst-like structures termed “human blastoids” were generated [152,154,156]. The blastoids are morphologically similar to blastocysts, with a TE-like outer compartment with apical–basal polarity and tight junctions that expresses TE markers. They also have a cavity and contain inner cell mass-like cell clusters that can further develop into EPI-like cells and primitive endoderm (PrE)-like cells [156].

It is possible to generate human blastoids not only from naïve hPSCs, but also from human expanded (extended) potential or pluripotent stem cells (hEPSCs) [155,157].

In a parallel study, it was found that, during the reprogramming of human fibroblasts into iPSCs in a 3D culture system, the cells aggregated and developed a cavity, consistent with a blastocyst-like structure [153]. This model of the human-induced blastocyst, termed iBlastoids, resembled the overall architecture of blastocysts, presenting an inner cell mass-like structure, with epiblast- and primitive endoderm-like cells, a blastocoel-like cavity, and a trophectoderm-like outer layer of cells. iBlastoids can give rise to pluripotent and trophoblast stem cells and TSCs are capable of expanded differentiation into EVTs and STBs [153].

Single-cell RNA sequencing analyses also reveal the transcriptomic similarity of blastoids to blastocysts. Unexpectedly, studies conducted by two groups also found a small subset of cells that appeared unique to the in vitro models and were uncommitted to any of the lineages and were of undetermined identity [152,153]. This could limit the use of this model if blastoids do not accurately recapitulate the cellular organization and lineage composition of the natural human blastocyst [158]. However, the gene expression profiles in blastoids derived by Kagawa and colleagues were found to be more similar to the EPI, TE, and PrE lineages in normal blastocysts and, furthermore, the sequence of lineage marker expression and the appearance of the EPI, TE, and PrE lineages corresponded to the same order in normal blastocyst development [156].

The last blastoid model was used for an in vitro study of implantation and revealed that the epiblast induces the local maturation of the polar trophectoderm and subsequently endows it with the capacity to attach onto hormone-stimulated endometrial cells, but not unstimulated ones [156]. After attachment to endometrial cells, the EPI-, TE-, and PrE-like cells continued to expand upon prolonged culture with the TE-like cells and formed trophoblasts expressing chorionic gonadotropin β, further differentiating into syncytio- and extravillous trophoblasts [156].

Due to the possibility of obtaining hundreds of these structures in one experiment, the “blastoid” system facilitated the study of early human development and the effects of genetic alterations or a toxic environment during early embryogenesis. These were the first in vitro models of human blastocysts and opened up possibilities for researching the causes of implantation failure and developmental defects at early stages of pregnancy.

### 5.7. Extended Blastocyst Culture

The first report of the successful growth of human blastocysts for extended periods was published in 2016, when a culture system supporting human embryo development up to day 13 postfertilization (the legal limit of human embryo culture) was approved and replicated the in vivo transition from pre- to postimplantation stages [159,160]. Several important discoveries were made, including the observation that human embryos show self-organization in the absence of maternal influences after attachment, with the generation of the embryonic and extra-embryonic germ layers, yolk sac, and amniotic cavities. Unlike in mice, human embryos have delayed ICM cell sorting and cell fate specification; moreover, human-specific cell populations were found, named the “yolk-sac trophectoderm” (ysTE), which is a transient embryonic tissue of trophectodermal lineage adjacent to the yolk sac [159,160].

Early establishment of TE territories and major morphological transformations of postimplantation human development, such as the differentiation of the trophoblast into the cytotrophoblast and syncytiotrophoblast, were demonstrated. Before blastocyst attachment (days 6–7), the TE of the cultured embryos expressed markers of early trophoblasts (CDX2, GATA3, and KRT7), with weak expression of OCT4 (pluripotency marker) and GATA6 (hypoblast marker), which declined to nothing after day 8. Most human blastocysts attached on days 7–8 at the polar TE, which is adjacent to the ICM. On day 10, some TE cells positive for GATA3 and KRT7 differentiated, with a subpopulation of cells arising that expressed human chorionic gonadotropin beta (HCG B), the STB marker, indicating the formation of the early syncytium. A layer of mononucleated cells adjacent to the substratum was also observed [159].

Interestingly, while both CDX2 and GATA3 are TE-specific markers, these studies have shown that GATA3 is a better marker of human TE because of its high signal and consistent nuclear localization, whereas CDX2 expression was less pronounced among TE cells [159,160]. Using a combination of extended culture with single-cell RNA-seq, the transcriptome dynamics in trophoblast cells occurring within the primitive placenta between day 8 and day 12 postfertilization were captured.

This culture method has been taken forward by the addition of a 3D matrix, which allows further development up to the gastrula stage. A 3D blastocyst culture system more accurately recapitulates in vivo conditions and provides the possibility of studying human embryo development beyond implantation [161]. Findings from this approach determined the developmental landmarks and 3D organization of human embryos, including the embryonic disc, amnion, basement membrane, primary and secondary yolk sac, formation of anterior–posterior polarity, and primitive streak anlage.

Based on extended 2D and 3D cultures, single-cell transcriptome and methylome maps of postimplantation human embryos were generated and the regulatory networks that underlie the segregation of the EPI, primitive endoderm, and trophoblast were delineated [161,162,163,164]. Zhou and colleagues identified genes that are specifically expressed in the human TE, EPI, and PrE lineages [162]. Simultaneous analysis of the gene expression network and lineage-specific DNA methylation patterns showed four major clusters, with a combination of the EPI, PE, and TE at the blastocyst stage (day 6) as a single cluster, and the EPI, PE, and TE beyond the blastocyst stage as another three separate clusters, suggesting that all three of the lineages underwent considerable changes in DNA methylation soon after implantation. Specific pathways and transcription factors triggering the postimplantation differentiation of CTBs, STBs, and EVTs were shown. These transcription factors included well-documented TB and pluripotency factors and new potential transcription factors, such as MYBL2, TCF7L1, and NR2F2 [161]. STBs on days 8–12 in the placenta are not equivalent to villous STBs, although they have an analogous function in supplying the embryo proper with nutrient support [163]. Additionally, the migratory trophoblast cells (MTBs) should probably not be referred to as extravillous TBs, since there are no villous structures at this stage from which MTBs could arise. It was found that initial implantation events and endocrine support of ovarian progesterone production by the mother are a function of the STBs surrounding the conceptus, while the motile MTB is responsible for initiating the additional colonization of the uterine endometrium prior to the outgrowth of placental villi. Transcriptome profiles of the MTB, including the expression of HLA-G, resemble those of first-trimester EVTs arising from the tips of anchoring villi [162,163].

Extended human blastocyst cultures establish a model system relevant to implantation failure and early human pregnancy loss studies, because the peri-implantation period is a crucial time in pregnancy, when placentation establishes communication between the mother and the implanting embryo. Using this platform, studies of the developmental consequences of chromosomal mosaicism or instability [165] and specific whole-chromosome aneuploidies [166] during the peri-implantation stage were recently published. Good-quality blastocysts were cultured in vitro up to day 12 and analyzed using high-resolution sequencing approaches. As seen in these studies, embryos with trisomies (collectively 15, 16, 21, and 22) all remained viable at day 12, while embryos with monosomies were significantly more likely to detach after day 8 [165,166]. In accordance with miscarriage tissue data [167], these findings suggest the predominant lethality of autosomal monosomies at the time of implantation or shortly thereafter. Similar outcomes were revealed for structural aberrations, with embryos bearing duplications more likely to develop up to day 12 compared to those with deletions [165]. Furthermore, all embryos diagnosed with multiple aberrations were arrested before day 12, attesting to the higher genetic burden of complex chromosomal abnormalities, while a high proportion (58%) of embryos originally diagnosed as mosaic remained viable at day 12 [165].

## 6. Availability and Advantages of Different Types of Cellular Material for Miscarriage Studies

Miscarriage most often occurs in the first trimester of pregnancy, and is apparently associated with hard disturbances of implantation and embryo–maternal interplay. Milder variants of abnormalities of the placenta extension will also have pathological manifestations later in development, such as preeclampsia or FGR. The direct study of placenta pathologies is often confused by an inability to determine whether placental abnormalities are a cause or a consequence of a given pregnancy complication. When placental tissues are obtained after a spontaneous pregnancy loss, the observed defects may be the result of gene mutations or an unbalanced karyotype in the embryo, or the effect of a maternal reaction to the presence of a nonviable pregnancy [168].

The use of cell and organoid models of trophoblasts for the study of miscarriage can be carried out in various directions: (i) forward, where cells obtained from the tissues of pregnancy loss with abnormalities responsible for embryo lethality are used for modeling; (ii) reverse, when specific mutations, probably resulting in miscarriage, are introduced into normal cells using genetic editing methods, followed by the modeling of specific aspects of trophoblast development. Therefore, various techniques for obtaining cell and/or organoid models of trophoblasts can be applied to simulate pathogenesis in specific disorders.

For direct studies, first-trimester miscarriage offers an unprecedented opportunity for access to human placental tissues, since the placenta at this period can be a source of TSCs [123]. It is promising to establish TSCs from a variety of genetic backgrounds associated with early miscarriage. Human TSCs are patient-specific, with preserved unique characteristics of trophoblastic cells, have a self-renewing capacity with a very long or indefinite replicative life, and can differentiate into various types of trophoblastic derivatives and, therefore, are very useful in analyses of the defects of placental development and function. The ability to create TSC lines from tissues obtained after early miscarriage poses no ethical problems and is an appropriate way to use these stem cell models to study the principles of early pregnancy loss. However, there are no experimental data on how long viable hTSCs persist in lost pregnancy material and in which specific context they may or may not be isolated. In their study of miscarriage, Saha and co-workers established TSCs from RPL placentae, and were able to derive TSC lines in most cases, but some RPL placentae, with a prominent defect in placental villi formation, had an extremely slow proliferation rate; two of the seven cases did not produce TSCs [8]. Given that some chromosomal or gene variants cause a decrease in or absence of proliferative activity of abortion cells in culture [47], the derivation of TSCs from the primary tissues of the placenta of some early spontaneous abortions may also be problematic.

Another source of human TSCs for miscarriage study is hPSCs, either naïve or primed (conventional). Naïve hPSCs are more closely related to early blastocysts and give hTSLCs that are fully consistent with primary (natural) hTSCs. However, issues related to the loss of epigenetic specificity, such as X chromosome inactivation or imprinting, may hinder the use of naïve hPSCs in the modeling of early placental disease [123]. Primed PSCs produce TSC-like cells of varying degrees of resemblance, depending on the method of obtaining them, which could also be more similar to amniotic or mesodermal cells [97,100,101]. Since hiPSCs can be derived by the reprogramming of easily accessible somatic tissues, they may provide a broad spectrum of patient-specific cells with a variety of genetic backgrounds, offering a way to uncover the genetic origins of common pathologies affecting the trophoblast lineage, such as miscarriage, pre-eclampsia, or FGR. However, it cannot be ruled out that the reprogramming of somatic cells can influence methylome establishment [169], so the variation of epigenetic signatures associated with placental pathology may restrict the usefulness of these models. Thus, further research is needed to determine whether hTSLCs derived from iPSCs associated with placental pathology will retain the disease phenotype. Nevertheless, stable, self-renewing isogenic human iPSC and iTSC lines provide an attractive opportunity to study human early development, and to better understand their roles in determining early gestation events that impact placental function. Another advantage of iPSC-based approaches is the possibility of obtaining material from cases with placental pathology and matched controls, which is not possible with other modeling approaches. Furthermore, hPSCs can undergo efficient genetic modification, which potentially enables the production of isogenic TSC lines with or without mutations, which is a very useful means of modeling normal and pathological trophoblast development. The advantages and limitations of stem cell-based trophoblast models for miscarriage research are given in Table 1.

## 7. Stem Cell-Based Studies of Chromosomal Aneuploidy and Mosaicism in Human Trophoblast Development

Aneuploidy, the presence of an abnormal number of chromosomes, is thought to be a major cause of human first-trimester pregnancy loss [11,13,14,44,45,46,48,173,174]. Aneuploidy rates are remarkably high in early human embryos, with more than 50% of IVF embryos diagnosed as aneuploid, including those from young, fertile couples [175,176,177]. Analysis of previously generated single-cell RNA-sequencing datasets with a total of 101 embryos [134,162] found an aneuploidy rate of 81.8% in preimplantation embryos at day 3, with a sharp decline starting from days 4–5, down to 5.4% at day 7 [119]. Embryos with nearly all variants of single-chromosome aneuploidies are viable until the blastocyst stage [5,167,178,179,180].

Some aneuploidies are compatible with live birth (trisomy 13, 18) and even with long-term postnatal development (trisomy 21, monosomy X, and sex chromosome trisomies), while others are incompatible with development from preimplantation up to clinically recognizable pregnancies (most autosomal monosomies, trisomies 1 and 19) [167]. However, having successfully passed through implantation, the vast majority of autosomal trisomies and polyploidies result in pregnancy loss.

It is suggested that aneuploidies have both direct (primary) effects, leading to alteration of the dose of genes localized on this chromosome, and also complex indirect (secondary) effects that implicate regulatory networks due to changes in the chromatin spatial interactions or TAD boundaries [181]. In addition, aneuploidy is known to cause proteotoxic, oxidative, and hypo-osmotic stresses and other nonspecific effects, decreasing the cell proliferation rate [182,183]. Comparative transcriptome analysis of IVF embryos with normal vs. abnormal karyotypes found that, while embryos with viable aneuploidy displayed transcriptomes that resembled those of normal embryos, blastocysts with nonviable aneuploidy had a large number of dysregulated genes, some of which showed a 100-fold difference in expression [184]. The degree of massive gene dysregulation of embryos with nonviable aneuploidies at the blastocyst stage suggested that the nonviability of the majority of chromosomal abnormalities is caused by very early events in development [185].

Although extensive research into human aneuploidy has shed light on the mechanisms of its occurrence [186,187], and manifestations in early embryogenesis [185,188,189], currently, there is only a superficial understanding of its influence on the further development of the embryo. Which defects of embryogenesis result in the lethality of embryos bearing specific aneuploidies remains unknown. It is likely that most peri-implantation and first-trimester embryo loss is caused by significant developmental abnormalities of the extra-embryonic lineage. Given the inaccessibility of human embryos at these stages of pregnancy, some issues concerning the developmental consequences of aneuploidy for human reproduction could be studied using trophoblast stem cell-based approaches and extended blastocyst cultures. Extended blastocyst cultures proved that embryos with trisomies are viable, while embryos with monosomies detach after day 8 [165,166], which is in accordance with the common absence of autosomal monosomy in miscarriage tissues, suggesting the predominant lethality of autosomal monosomies at the time of implantation or shortly thereafter.

While whole-organism aneuploidy is the most common cause of embryo death leading to miscarriage, aneuploidies in some cells (chromosomal mosaicism) may not have a noticeably negative effect or, in some cases, may even confer an advantage. Recent technological advances have provided evidence that a significant proportion of early human embryos are mosaic. Estimating mosaicism in human embryos depends on the developmental stage and testing technique, and is influenced by the number of cells analyzed. Thus, using an NGS assay, blastocyst mosaicism reported based on a single TE biopsy has been described as affecting 2–13% of the embryos tested [190,191], while studies disaggregating whole embryos suggest that mosaicism may be present in up to 50% of blastocysts [17,192,193,194]. Recent studies on single-cell transcriptomic data revealed widespread mosaic aneuploidies, with 80% of embryos bearing at least one putative aneuploid cell [195] and highly variable proportions of aneuploid cells per embryo [119]. Aneuploid cells were found to be most abundant in mosaic embryos in the early stages, while declining over time [119]. Recent findings revealed that mosaicism is a typical feature of placental development due to extensive mutagenesis in placental tissues [196].

One of the most intriguing questions currently being addressed by research is the selective elimination of aneuploid cells, suggested by normal live births after the intrauterine transfer of mosaic aneuploid embryos [16,20]. Experiments on extended blastocyst cultures showed that a high proportion (71%) of embryos originally diagnosed as mosaic were found to be euploid [165]. Mechanisms of embryo karyotype normalization are under discussion. Daughtry and colleagues used a nonhuman primate model with an aneuploidy rate similar to that in humans to examine the ability of embryos to overcome chromosome instability during preimplantation development. It was found that chromosomal errors were corrected by i) encapsulation into micronuclei, ii) elimination of abnormal blastomeres via cellular fragmentation, and iii) selection against highly aneuploid blastomeres [197]. In human embryos, whole-genome amplification of the blastocyst and its corresponding debris followed by aCGH found that 63.6% of blastocysts expelled cell debris with additional chromosomal rearrangements, and 55.5% of euploid blastocysts expelled aneuploid debris [198]. Additionally, considerably higher levels of cell proliferation and death were revealed in mosaic and aneuploid blastocysts compared to their euploid counterparts, which supported the hypothesis of the self-correction of the embryo karyotype [199]. An increased number of chromosomal abnormalities in the blastocoel fluid, depending on the degree of maturity of the blastocyst from stage 3 to 5 of blastulation, indicated the more intensive elimination of cells with chromosomal aneuploidies during this period [200]. Single-cell sequencing data from human embryos indicated that the negative selection process against aneuploid cells intensifies during postimplantation development [195]. Collectively, these observations indicate that mosaic aneuploidies can be subject to negative selection and actively or passively eliminated from the conceptus throughout development.

Chimeric mouse embryos consisting of euploid and aneuploid cells and followed by single-cell tracking were used to address the embryo’s capacity for self-correction in different lineages. It was found that aneuploid cells in the epiblast have a higher rate of apoptosis than aneuploid cells in extra-embryonic tissues, which are tolerant of aneuploidy but show proliferative defects [201,202]. The expression of proapoptotic genes was upregulated in early postimplantation mouse epiblast cells, which led to a lower apoptotic threshold in embryonic versus extra-embryonic cells in response to DNA damage [203,204].

Recently, Yang and co-workers tested whether there was the same preferential depletion of aneuploid cells in the human epiblast as in mouse models of mosaic aneuploidy [119]. The fate of aneuploid cells in mosaic human embryos was simulated using reversine-treated “gastruloids” and it was found to be quite specific [119]. As a result of BMP4 treatment, aneuploid gastruloid colonies mainly produced GATA3-positive cells due to the predominant survival of extra-embryonic tissue, while SOX2-positive (ectoderm) and BRA-positive (mesoderm) domains were largely absent, with an increase in cell death marker CASP3. The cell death during “gastruloid” formation was found to be a consequence of the BMP-induced differentiation of aneuploid pluripotent cells, but the exact molecular mechanisms of this phenomenon still need to be explored. An interesting speculation about the mechanism of selective cell death was proposed based on interspecies PSC co-culture experiments: highly proliferative perigastrulation epiblast cells (which correspond to primed PSCs) may have evolved a mechanism, putatively through the innate immune system (Toll-like receptors (TLRs)/Myd88), to activate nuclear factor kappa B (NFκB)-dependent apoptosis in “aberrant” or “unfit” cells, thereby preventing them from further participation in development [205]. This mechanism could account for the lineage preference in aneuploidy-induced cell death during early cell fate specification.

It is noteworthy that among the top genes with significant cell-type-specific responses to aneuploidy was the transcription factor GATA3, a known trophectoderm lineage marker with a role in promoting trophectoderm fate [206]. GATA3 exhibited no significant response to aneuploidy within the trophectoderm, but downregulation was seen in aneuploid cells of the ICM and descendant epiblast [195]. Interestingly, GATA3 was significantly upregulated in undifferentiated aneuploid cells of cleavage-stage embryos, thus enabling researchers to speculate that aneuploidy itself may bias lineage decisions [195].

These studies highlight the possibility of different responses to aneuploidy in embryonic and extra-embryonic tissues, at least at some developmental stages. Thus, the “clonal depletion hypothesis” seems to be more accurate as a mechanism behind the survival of mosaic embryos than the “self-correction” of chromosomal abnormalities [207]. Whole-genome sequencing revealed that developmental bottlenecks can genetically isolate trophectodermal lineages from lineages derived from ICM, thus promoting the normalization of zygotic aneuploidy [196]. Whether the different responses to aneuploidy in embryonic vs. extra-embryonic tissue reflect cell-type-specific apoptosis and/or proliferation defects of aneuploid cells is an open question for future study [208].

If aneuploid cells in mosaic embryos tend to survive in the trophectoderm, with depletion in ICM, this could explain the prevalence of confined placental mosaicism (CPM) at later developmental stages; it occurs in at least 2% of ongoing natural pregnancies [209,210,211], and in 3.5% of miscarriages [48]. It was found that, in some cases, CPM is not associated with adverse pregnancy outcomes [212,213], but other authors reported that pregnancies complicated by CPM were associated with an increased risk of intrauterine growth restriction or intrauterine fetal death [24,214], preterm birth, and small-for-gestational-age newborns [215,216,217].

Nevertheless, aneuploidy in the extra-embryonic lineage should not necessarily be viewed as abnormal. Despite the detrimental effects of massive gene dosage imbalances, changes in karyotype appear to confer proliferative advantages in certain circumstances. It is known that aneuploidy is found at a high rate both in cancer cells [218], providing a proliferative advantage, and in some normal specialized cells—for example, in the liver and the brain—which suggests that aneuploidy may be a source of genetic variation [219]. The tolerance towards chromosome instability in extra-embryonic tissue, similar to that seen in cancer, might be associated with the rapid proliferation, migration, and deep trophoblast invasion required for the success of human pregnancy, suggesting the possibility of a functional value for aneuploidy in the placenta [119,127,220,221]. Thus, an unexpectedly high rate of aneuploidy (more than 95%) among the human CTB subpopulation that exits the cell cycle and invades the uterus was found, suggesting that these aberrations in chromosome number are a normal part of CTB differentiation rather than an anomaly [220]. Recently, reconstruction of the somatic genetic architecture of human placentae using whole-genome sequencing revealed the clonality of placental tissues and a comparatively high mutation rate with, uniquely for non-neoplastic human tissue, frequent copy number changes, similar to some types of human tumors [196].

Single-cell data from an extended blastocyst culture revealed that both meiosis- and mitosis-derived CNVs were widely present in cultured embryos during implantation, and these aneuploid cells clustered with the corresponding euploid cells, suggesting that the differentiation of the major lineages was generally not distorted by mild CNVs at the early stage of implantation [162]. Kasak and co-workers detected an extensive load of CNVs in human placentae and hypothesized that, similarly to the situation in cancer, somatic genomic rearrangements promoted the placental function [221]. However, some recent results contradict this view, since placental de novo CNVs were found in only 10.8% of families, and enrichment analyses of genes located in these regions did not reveal any molecular pathway related to placental biology, indicating that these CNVs are probably sporadic and do not play a role in the function of the placenta [222]. Further studies of trophectodermal and trophoblast cells in model systems in vitro will help to clarify the prevalence, versatility, and underlying mechanism of the tolerance towards chromosome instability in extra-embryonic tissue.

Building stem cell models of aneuploid embryonic and extra-embryonic cells will be useful in the investigation of the mechanisms underlying the different developmental consequences of specific aneuploidies. The research by Grati and colleagues offers comprehensive recommendations by determining specific risk scores across individual chromosomes, based on chorionic villus sampling and the analysis of miscarriage samples [223]. The risk scores for each mosaic aneuploidy rely on the likelihood that the mosaic aneuploidy is also present in the fetus and that it results in clinically significant fetal UPD. However, placenta-specific manifestations of aneuploidy may also be a risk factor for pregnancy complications [224]. Thus, information about the placenta-specific manifestations of aneuploidy may be important for the decision on the transfer of mosaic embryos. Considering that, in mosaic embryos, aneuploid cells are more often concentrated in the extra-embryonic lineage, modeling of the stem cells of trophoblasts will make it possible to establish which specific aneuploidies have a detrimental effect on the function of the placenta and, accordingly, a mosaic embryo with this chromosome may be assigned lower priority [217].

Trisomy 16 is one of the most studied aneuploidies, with the highest rate found in spontaneous abortion. Extended embryo culture showed that trisomy 16 embryos display a strong hypoproliferation of the trophoblast [166]. Using TSCs and ESCs as models of the trophoblast and epiblast, respectively, researchers showed that poor trophoblast proliferation can be mechanistically attributed to increased levels of a gene(s) located in chromosome 16, specifically *CDH1*, which encodes the cell–cell adhesion protein E-cadherin. Increased E-cadherin levels resulted in cell cycle arrest and differentiation in TSCs, while ESCs remained pluripotent [166]. Interestingly, in the placentae of first-trimester spontaneous abortions with trisomy 16, hypermethylation of promotor and expression downregulation of trophoblast marker GATA3 were found [225], which is in agreement with extended blastocyst culture findings.

The predominance of the meiotic origin of trisomy 16 in embryos [187], together with its adverse impact on early placental development, explain why it is one of the most frequently observed genetic abnormalities in spontaneous abortions up to 10 weeks of gestation [48], but is less commonly detected in noninvasive prenatal testing that is usually performed at week 12 [226,227]. The inappropriate trophoblast development observed in trisomy 16 embryos could potentially explain the intrauterine growth restriction and preeclampsia commonly observed in cases of CPM of trisomy 16 [228].

Maxwell and colleagues used NGS to reanalyze TE biopsies originally classified as euploid by aCGH, and found that 32% of the blastocysts that miscarried in pregnancy were mosaic [229]. This was the first evidence that the diagnosis of mosaicism may be associated with early pregnancy loss. Compared to euploid embryos, mosaic embryo transfer was associated with an increased risk of miscarriage in multiple studies [179,230,231,232]. At the same time, some studies reported that transfers of chromosomally “abnormal” embryos resulted in ongoing pregnancies or live births, with low (9.3%) miscarriage rates [18]. However, in such cases, caution is necessary, since autosomal aneuploidy was over 10 times more frequent in SGA-associated placentae compared to controls [216] and CPM is a high-risk condition when chromosomes 2, 3, 7, 13, 15, 16, or 22 are involved [217].

## 8. Future Perspectives

The growth in the number of platforms to model trophoblastic lineage using stem cell-based technologies that we are seeing now should, in the near future, lead to an increase in the number of studies on the functional consequences of genetic variants for placental development. In vitro modeling enables us to reveal the role of specific genes and chromosomal aberrations in both the self-renewal and differentiation of TE/TSCs and investigate the molecular basis of pregnancy complications, for which failures of trophoblast growth and differentiation could be underlying causes. This technology could be greatly advanced by integrating it with the possibility of the genetic manipulation of human stem cells [233] as well as human embryos [234,235,236]. In addition, methods are being developed for the correction of large-scale chromosomal abnormalities, including CNVs and numerical aberrations, in PSCs [237,238].

Owing to the advent of high-throughput technology, scRNA-seq is enabling single-cell transcriptome analysis at high resolution and will contribute to our understanding of the molecular-level regulatory mechanisms underlying early human placenta development as well as placenta-associated pregnancy complications. Modeling human trophoblast development using state-of-the-art technologies will facilitate the creation of a reference atlas across gestation, the discovery of new cell types, and the dissection of the cell–cell interactions [8,134,146,161,162,239,240]. As with any model, hTSCs in vitro do not accurately render some details of in vivo differentiation and tissue development—for example, the polyploid phenotype seen in primary EVTs [127]. This limitation calls for the creation of more optimized in vitro models that simulate inter-tissue signaling involving growth factors, hormones, cytokines, and others.

Since 3D cultured cells more accurately recapitulate authentic tissue environments, further development of trophoblast organoids will enable us to study the mechanisms behind the communication of multiple trophoblast cell types [142,143,144]. In addition, crosstalk between the endometrium and trophoblasts will be elucidated using endometrial organoids [241,242]. In particular, the combination of organoid technologies that mimic the development of embryos, trophoblasts, and the endometrium has the potential to replicate the complexity of the maternal–fetal interface more precisely [241,243,244].

The system of self-assembling artificial human embryos called “blastoids” facilitates the study of early human development in the context of the effects of different genetic alterations due to the possibility of obtaining hundreds of these structures from one experiment [152,153,154,155,156,157]. In addition, many other factors can be explored, such as the effects of pathogens, hormones, immunity, or a toxic environment during early embryogenesis. Thus, human blastoids may be used to identify therapeutic targets and contribute to modeling the causes of implantation failure or embryo rejection at the early stages of pregnancy. For example, in the study by Kagawa and colleagues [156], blastoids only attached to hormone-stimulated endometrial layer cells but not to unstimulated ones, which enables implantation research that could open the “black box” of human implantation [1].

## 9. Conclusions

Several decades of mainly descriptive research on the genetic basis of miscarriage have produced a bulk of knowledge about chromosomal abnormalities, microstructural rearrangements, and gene variants, as well as epigenetic disorders, leading to embryonic death and miscarriage. Now, research in this area is entering a fundamentally new phase of experimental studies, wherein the underlying molecular mechanisms of the pathogenesis of pregnancy loss can be established by using techniques based on stem cells to construct in vitro models of human development. This review considers the most relevant in vitro systems of trophoblast stem cells and organoids, human blastoids, and extended embryo cultures, and describes the most significant experiments related to human miscarriage.

We highlight a number of important issues in human reproduction that might be addressed by such techniques.

(i) The influence of specific aneuploidies and gene variants on embryonic development. Each specific abnormality involves its own risk of embryonic arrest or failure to implant, spontaneous abortion, or abnormal live birth. hTSCs and organoids have already been successfully used to study gene function or manifestations of chromosomal abnormality in human trophoblast lineages. Large coordinated databases can be created to further study the risks associated with each aneuploidy and gene variant.

(ii) The self-correction ability of the human preimplantation embryo. Human preimplantation development is remarkably prone to mitotic error, so mosaicism is found frequently in human embryos. Human “gastruloids,” artificial embryo-like blastoids, and extended embryo cultures provide the tools to explore, manipulate, and mimic human embryo development in a dish, thus opening up a new avenue to research the developmental capacity of mosaic blastocysts, the distribution of aneuploid cells between epiblasts and TEs, and the selective death or clonal depletion of aneuploid cells.

(iii) The impact of a specific mosaic aneuploidy, especially confined placental mosaicism (CPM), on the subsequent developmental potential of the fetus. After PGT-A, sometimes only mosaic embryos are available, and when an anomaly is found only in the trophectoderm, it can lead to CPM formation. A better understanding of the underlying biological mechanisms behind CPM will help with the selection of embryos for transfer after the detection of mosaicism.

In conclusion, trophoblast stem cell technologies allow us to research patient-derived and genetically manipulated cells, which can be cultured long term and biobanked, produce multiple trophoblast cell types, and provide a powerful model to understand human trophoblast development, including the pathogenesis of pregnancy loss.

## Figures and Tables

**Table 1:** Advantages and limitations of stem cell-based trophoblast models.

Stem Cell-Based Model	Cell Source	Advantages	Limitations	Key Achievements of Model Application for Genetics of Miscarriage or Trophoblast Dysfunction Pathology
BMP4-induced trophoblast-like cells	ESCs [93,94]; iPSCs [95]; PSCs [96]	Availability of source material; possibility of genetic modification; variety of genetic backgrounds	Lacking self-renewal; expression does not fully correspond to trophoblasts	Trisomy 21: delay in the induction of the trophoblast lineage, lower fusion index, increased hCG secretion [96]
				Monosomy X: altered levels of placental genes; skewed trophoblast cell type composition [103]
				Translocation (11;22): impaired expression of trophoblastic genes; lower invasiveness [108,112]
				Preeclampsia: reduced trophoblast invasion under high O_2_ conditions [115,116]
				Recurrent complete hydatidiform mole: precocious downregulation of pluripotency factors and activation of trophoblast lineage markers [84]
				Lineage-specific behavior of aneuploid cells in “gastruloids” with prevalent survival of extra-embryonic tissue [119]
“True” human trophoblast stem cells (hTSCs)	Blastocysts; 1st trimester placentae [123]; term placenta [170]	Patient-specific; preserved characteristics of trophoblastic cells, unlimited self-renewing capacity	Derivation from the primary tissues of some abortions may be problematic	Association of idiopathic RPL with altered expression of TEAD4; imbalance of self-renewal vs. differentiation in idiopathic RPL placentae [8]
				Complete hydatidiform mole exhibits resistance to contact inhibition [171]
				Neddylation inhibition hindered EVT differentiation [125]
				Depletion of MSX2 resulted in precocious STB differentiation [172]
				Decreased CKMT11 expression associated with PE [170]
Trophoblast stem-like cells (TSLCs)	Naïve PSCs [97,98,124,128]; primed PSCs [89,90,100,136]; EPSCs [135]	Availability of source material; possibility of genetic modification; variety of genetic backgrounds	Possible inconsistencies of epigenetic specificity	No data currently available
Induced trophoblast stem cells (iTSCs)	Somatic cells [137,138]; term placenta vCTBs [141]			
3D culture models	1st trimester CTBs [142,143,144]	More authentic tissue environments; self-renewing ability	Limited self-renewal in some studies	Treatment with YAP/TAZ inhibitor reduced organoid growth and expression of cyclin A [147]
	hTSCs [8]			Inefficient organoid formation for RPL cases with reduced expression of TEAD4 [8]
				Transcriptomic landscape for CTB commitment to EVT or SCT; BCAM is a primitive progenitor marker [146]
	Naïve PSCs [145]			Dynamics of X chromosome inactivation [145]
Blastoids	Naïve PSCs [152,154,156]	A large number of units in one experiment; availability of source material; possibility of genetic modification; variety of genetic backgrounds	Expression discrepancy with blastocysts in some models	No data currently available
	hEPSCs [155,157]			
iBlastoids	Fibroblasts [153]			
Extended blastocyst culture	Blastocysts [159,160]	Patient-specific; preserved native characteristics of cells; autonomy from maternal contribution	Limited access to source	Lethality of autosomal monosomies and multiple aberrations at the peri-implantation period; embryos with duplications develop longer than with deletions [165,166]
3D extended blastocyst culture	Blastocysts [161]			
